# Bypassing Heaven’s Gate Technique Using Black-Box Testing

**DOI:** 10.3390/s23239417

**Published:** 2023-11-26

**Authors:** Seon-Jin Hwang, Assem Utaliyeva, Jae-Seok Kim, Yoon-Ho Choi

**Affiliations:** School of Computer Science and Engineering, Pusan National University, Busan 609-735, Republic of Korea; unlockable@pusan.ac.kr (S.-J.H.); assemakx@pusan.ac.kr (A.U.); tjr3117@pusan.ac.kr (J.-S.K.)

**Keywords:** malware detection, anti-debugging, bypassing anti-debugging

## Abstract

In recent years, the number and sophistication of malware attacks on computer systems have increased significantly. One technique employed by malware authors to evade detection and analysis, known as Heaven’s Gate, enables 64-bit code to run within a 32-bit process. Heaven’s Gate exploits a feature in the operating system that allows the transition from a 32-bit mode to a 64-bit mode during execution, enabling the malware to evade detection by security software designed to monitor only 32-bit processes. Heaven’s Gate poses significant challenges for existing security tools, including dynamic binary instrumentation (DBI) tools, widely used for program analysis, unpacking, and de-virtualization. In this paper, we provide a comprehensive analysis of the Heaven’s Gate technique. We also propose a novel approach to bypass the Heaven’s Gate technique using black-box testing. Our experimental results show that the proposed approach effectively bypasses and prevents the Heaven’s Gate technique and strengthens the capabilities of DBI tools in combating advanced malware threats.

## 1. Introduction

As the Internet of Things (IoT) has grown in popularity and has become more commonplace in our daily lives, sensor devices have become increasingly prevalent. However, with the increased use of these devices, there is an increased risk of malware attacks [1,2,3] that continuously devise new methods to evade detection. In the evolving technological environment, malware authors adapt by crafting sophisticated tools and techniques to circumvent security measures [4,5,6]. This behavior delays the analysis time required by security analysts and enables adversaries to extend the period between initial release and detection. Malware authors employ virtualization, obfuscation, and anti-debugging techniques to achieve this purpose.

A recent investigation by Cisco Talos uncovered a unique malware campaign that included the HawkEye Reborn keylogger, among other malicious software [7]. This campaign highlights the clever tactics that cybercriminals use to avoid antivirus detection. One of the most notable features of this operation is the loader’s use of the ‘Heaven’s Gate’ technique. This approach allows 32-bit malware to bypass detection by hiding Application Programming Interface (API) calls and switching to a 64-bit execution environment.

This technique, known as the Heaven’s Gate, emerged with the introduction of the 64-bit computing environment. A 64-bit operating system should support 32-bit applications, considering that many legacy systems, hardware, and software are based on the 32-bit architecture. For instance, the Windows operating system includes the Windows-on-Windows 64-bit subsystem (WOW64) to execute 32-bit applications within a 64-bit architectural environment [8].

Although this technique is not inherently malicious itself, it has been exploited by malware authors to achieve malicious code execution without detection. By switching to 64-bit mode within a 32-bit process, malware can evade detection mechanisms that exclusively focus on inspecting 32-bit code. Consequently, this obfuscation renders it challenging for both static and dynamic analyses to identify malicious activities. For example, in static analysis, an actual 64-bit code is misidentified as a 32-bit code, leading to both false positives and false negatives. In dynamic analysis, although the actual 64-bit operation code should be executed during the analysis, the 32-bit operation code is executed, resulting in errors. Generally, a debugger, disassembler, or dynamic binary instrumentation (DBI) can be employed for malware analysis. These analysis tools are specifically designed for either 32-bit or 64-bit applications, and thus, for the analysis of 32-bit applications, a corresponding 32-bit analysis tool should be used. Therefore, they lack a countermeasure against Heaven’s Gate [9,10,11,12].

It is difficult to simply detect and block the operation of Heaven’s Gate because it is a technique used in Windows dynamic linked library (DLL) [13] and is also used in code protectors to prevent reverse engineering for protecting intellectual properties. For example, the latest version of VMProtect [14], a well-known tool for code protection, uses Heaven’s Gate as its default protection feature for 32-bit programs. Since malware can also use VMProtect including Heaven’s Gate, the misuse of the Heaven’s Gate technique is anticipated to increase.

Furthermore, the Heaven’s Gate technique was initially introduced in the 2010s, and recent reports indicate that this security threat persists [15,16,17,18]. Despite the sustained prevalence of the Heaven’s Gate technique, a significant research gap exists in the domain of automated analysis methods. Specifically, established approaches such as dynamic binary instrumentation and scripting techniques have yet to be extensively discussed or adopted [19,20,21,22]. It challenges existing malware analysis and defense strategies, necessitating advancements in cybersecurity tools and methods. At the same time, it presents an opportunity for malware authors to develop more sophisticated and hard-to-detect malware, underlining the importance of continuous evolution and adaptation in cybersecurity measures.

The limitations of the current research highlight the pressing need for advanced, automated methodologies that can effectively analyze and counter Heaven’s Gate-based anti-debugging techniques.

In this paper, we address the aforementioned research gap by introducing a novel automated method to analyze malware that employs the Heaven’s Gate technique. To understand how Heaven’s Gate works, we first analyzed the structure to which Heaven’s Gate is applied to understand its principles in a high-level code. We also proposed a novel method to bypass Heaven’s Gate using black-box testing and support it with an experimental evaluation that shows that the proposed method can successfully bypass the Heaven’s Gate technique in the latest version of VMProtect. To the best of our knowledge, this is the first study to bypass Heaven’s Gate for automated tools such as the DBI or script. The main contributions of this paper can be summarized as follows:

- We analyzed Heaven’s Gate execution from a low-level assembly code and a high-level C/C++ code.
- We proposed a method to bypass Heaven’s Gate and demonstrated dynamic binary instrumentation-based bypass in practice.
- We evaluated the effectiveness of the proposed method on a dataset comprising the latest version of VMProtect, confirming its practical applicability. We release the source code of our sourcecode and benchmark dataset at (https://github.com/unlockable/Bypassing-Heaven-s-Gate, accessed on 23 November 2023).

The rest of the paper is organized as follows. Section 2 provides background information about the Heaven’s Gate technique, while Section 3 describes related work. In Section 4, we first comprehensively analyze the Heaven’s Gate technique and describe the proposed method in Section 5. The experimental results are briefly described in Section 6. Finally, Section 7 and Section 8 conclude the paper.

## 2. Background

The term Heaven’s Gate is a metaphorical expression symbolizing a ‘gateway’ from 32-bit execution to 64-bit execution. This technique has been employed by malware authors to facilitate the transition from 32-bit to 64-bit code during the execution of a malicious payload. The technique that emerged in the 2010s enables 32-bit malware to switch to 64-bit code, bypassing security mechanisms and avoiding detection. The Heaven’s Gate technique enables the execution of 64-bit instructions, effectively transforming the corresponding x86 code into obfuscated “dummy” code for the purposes of misdirection.

Switching is achieved by manipulating the CPU’s segment selector from the 32-bit mode (code segment (CS) selector = 0x23) to the 64-bit mode (CS selector = 0x33) during the low-level bytecode execution. It is not a standard or recommended approach used by operating systems; rather, it is primarily employed to safeguard either malicious code or code associated with the protector. CS selector can be manipulated via three x86 assembly instructions: “jmp far”, “call far”, and “ret far”.

Once in the 64-bit mode, malware can access 64-bit system libraries and syscalls within a 32-bit process. This could enable malware to use techniques or attack surfaces that are not as closely monitored in the 32-bit mode. The complexity of switching between 32-bit and 64-bit codes can be used to obfuscate the true purpose of the code, making static analysis more challenging, as described in Listings 1 and 2. The more convoluted and confusing the code is, the more likely it is to slip undetected through static analysis.

**Listing 1.** 32-bit assembly analysis.

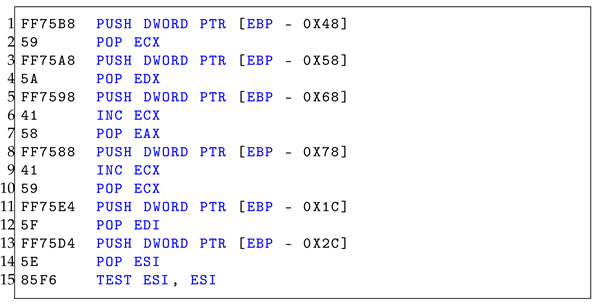



**Listing 2.** 64-bit assembly analysis.

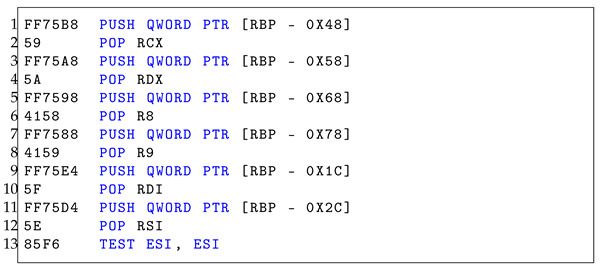



Listings  1 and 2 have the same byte sequence as Heaven’s Gate. Although the assembly instructions appear similar, they differ based on the manner in which the bit modes are applied. As demonstrated in Listing 1, the register and pointer operations employ 32-bit registers and double words. On the other hand, in Listing 2, 64-bit registers and quad words are employed. Furthermore, the bytecodes 0x41 and 0x58 in the 64-bit assembly (Listing 2, line 6–line 7) are interpreted as POP R8 and POP R9, respectively. However, in the 32-bit assembly (Listing 1, line 6), the 0x41 byte is interpreted as INC ECX. This difference arises because the 0x41 byte in the 64-bit mode is a prefix that extends the opcode to specify the use of additional registers (R8-R15) available in the 64-bit mode. In the 32-bit mode, 0x41 is the opcode for INC ECX. This implies that identical byte codes may yield different outcomes when executed in different environments.

Since a 32-bit program executes bytecodes using 32-bit instructions only, almost all debugging and disassembly engines employ distinct engines for each instruction, which results in errors or inconsistencies in the analysis. For example, to analyze a 32-bit application, the debugging engine predominantly uses the x86 instruction set to emulate the program’s behavior, whereas for 64-bit analysis, it relies on x86-64 instruction sets.

Heaven’s Gate can be applied to 32-bit programs in various 64-bit operating system environments [23,24]. For example, Microsoft has a compatibility technology known as WoW64 (Windows 32 in Windows 64) to run a 32-bit portable executable (PE) format in a 64-bit Windows environment [25].

## 3. Related Work

Several studies have been designed for anti-debugging techniques [19,22,26,27] which obstruct the effective analysis of malware through debugging tools, and for unpacking techniques [20,22,28,29] which involve extracting the original code from encrypted or packed executables.

Representative anti-debugging and unpacking techniques such as PinDemonium [20] and Arancino [22] leverage DBI, particularly those based on the Intel PIN. PinDemonium finds the original entry point (OEP) using heuristic methods such as entropy analysis and restores the import address table (IAT) using Scylla [30]. Additionally, PinDemonium solves the stolen API technique using the proposed IAT deobfuscation algorithm, while Arancino addressed anti-instrumentation techniques. Arancino’s unpacking features, such as the OEP search and IAT reconstruction, are very similar to those of PinDemonium.

Shi et al. proposed a framework called Apate [26], which systematically explored possible anti-debugging techniques and enumerated a comprehensive list of 79 distinct attack vectors. Apate was implemented as an extension of WinDbg and was extensively evaluated using five different datasets comprising both known and new malware samples. Lee et al. analyzed the techniques used by five of the most common commercial protectors [19]. They proposed a method to bypass anti-VM and anti-DBI techniques supported by commercial protectors through a detailed algorithm analysis.

Kim et al. investigated anti-analysis techniques used in real-world malware [31]. They assessed the frequency of anti-analysis techniques in Windows malware and designed an automated system, EvDetector, which identifies malware using these techniques and monitors changes in execution paths resulting from the anti-analysis strategies. Choi et al. devised a novel dynamic analysis framework for malware, called HybridEmu [32]. HybridEmu includes the automatic detection and evasion of multiple anti-reversing techniques. HybridEmu integrates a CPU simulator for machine instruction simulation with the direct execution of API functions upon their invocation. Park et al. introduced an automatic anti-debugging detection and bypassing scheme utilizing the Pin [27]. To assess their algorithm’s effectiveness, they tested it against 17 of the most widely used commercial protectors, successfully bypassing all anti-debugging techniques automatically.

BinUnpack [29] improved the OEP search algorithm based on Arancino by reducing the search scope. It also utilizes an API monitor module via a novel kernel-level dynamic link library (DLL) hijacking technique to reconstruct the IAT. BinUnpack has been extensively evaluated on more than 238,000 malware samples. Meanwhile, Choi et al. implemented x64Unpack [28], a hybrid application emulator for 64-bit portable executable files. They successfully decoded obfuscated API calls based on the Bochs emulator [33] and using the Run-Until-API method [34]. Table 1 summarizes the characteristics of each related study and how they differ from our study.

Unfortunately, PinDemonium remains the only open-source solution available for generic binary unpacking. Since reproducing these solutions is challenging owing to the extensive scope of the source code implementation, follow-up research has been difficult. For instance, x64Unpack implements 196 API functions for unpacking, and API-Xray implementation requires deploying a hardware-assisted mechanism and multiple components at both the kernel and user levels.

## 4. Analysis

To understand the Heaven’s Gate technique used by malware, we analyzed its high-level implementation in C/C++ programming languages. We chose an open-source implementation [23] that is susceptible to misuse by malware authors. It uses Heaven’s Gate to switch from a 32-bit to a 64-bit environment to execute the NTerminateProcess function.

Listing 3 represents its main function. For ease of explanation, we extracted key parts of the code from the existing source and omitted irrelevant parts. In this sample, the process is terminated by the Heaven’s Gate technique. It retrieves the 64-bit “ntdll.dll” module address by using the user-defined function GetModuleHandle64 (line 4). Next, the program verifies the validity of the 64-bit module address (line 5) and retrieves the address of the 64-bit NtTerminateProcess function using the user-defined function GetProcAddress64 (line 7). Finally, it calls the 64-bit NtTerminateProcess function using the user-defined function CallFunction64 (line 8–line 10). The CallFunction64 function uses the process ID and the 64-bit NtTerminateProcess address obtained as arguments.

The core operation of the Heaven’s Gate technique is executed within the CallFunction64 function (line 10), and the detailed implementation is provided in Listing 4. The function requires five parameters: (1) ‘ulAddress’, which specifies the 64-bit address of the function; (2) ‘ulOrdinal’, designated for system call tables; (3) ‘pulArgs’, serving as the 64-bit arguments for the function; (4) ‘ulArgCount’, the number of arguments for the function; and (5) ‘ucType’, a flag used to determine whether to initiate a system call or execute a normal function call. As mentioned in Listing 3, the variable ulAddress has the 64-bit address of the NtTerminateProcess function. The variable ulOrdinal has a value of 0, which appears erroneous given that the actual system call table entry for ‘NtTerminateProcess’ is 0x2c. 1-d. The array pulArgs contains the process ID to be terminated, and the variable ucType has a value of 0, indicating that it is a system call.

**Listing 3.** Main function of Heaven’s Gate.

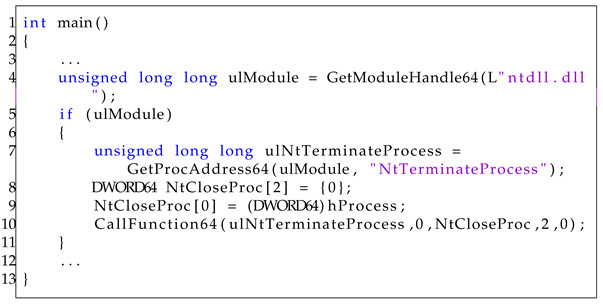



At the beginning of the CallFunction64 function in Listing 4, the variables are initialized (line 3–line 12), and arguments in the pulArgs array are parsed through a function composed of pointer operations (line 9–line 19). The remaining parts, corresponding to the core functionality of the Heaven’s Gate, comprise an inline assembly code. This includes not only 64-bit codes but also 32-bit codes. Initially, the current values of the EDI and ESI registers are pushed onto the stack (line 22–line 23). The values of StackArguments and StackCount are loaded into the registers rdi and rsi (line 34–line 36).

Next, argument values are assigned to each register, RCX, RDX, R8, and R9 (line 26–line 32), corresponding to the calling convention of x64 [35]. To use 64-bit registers, the function X64_Start is called, as shown in Listing 5. It consists of a brief sequence of four assembly instructions. It switches to a 64-bit context through the “retf” instruction by modifying the code segment (cs) value to 0x33.

If there are more than four arguments, additional arguments are assigned on the stack (line 39–line 42). Then, if the value of ucType is 0, it executes a normal function call. Otherwise, it executes a system call (NtTerminateProcess) in a 64-bit context (line 43–line 60). Finally, it switches to a 32-bit context by calling the X64_End function as the inverse of X64_Start function, as shown in Listing 6.

From the analysis, it is shown that Heaven’s Gate was used to hide certain functions. This was accomplished through 64-bit assembly instructions that a 32-bit disassembler engine could not execute.

**Listing 4.** CallFunction64 function source code.

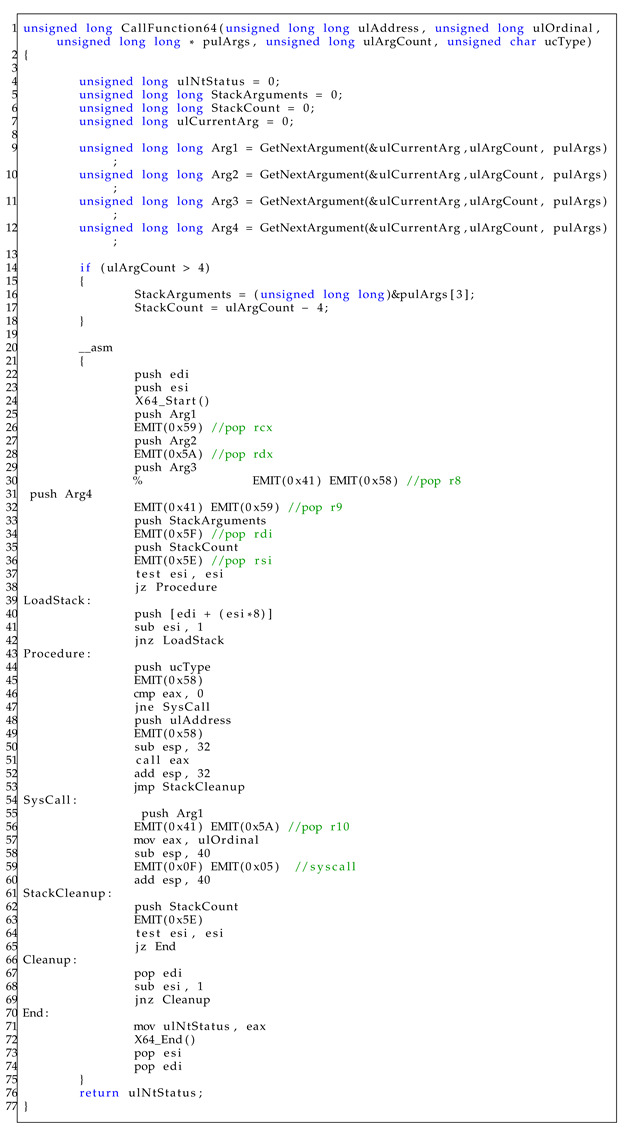



**Listing 5.** x64 start function.

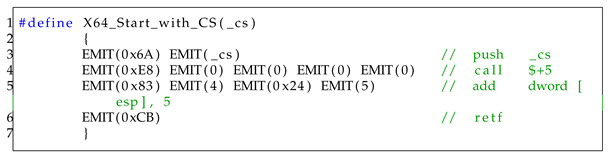



**Listing 6.** x64 end function.

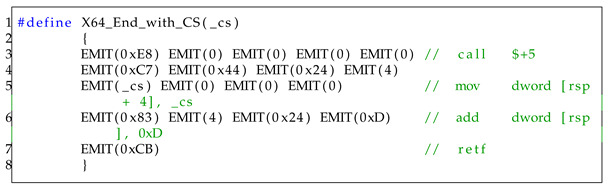



## 5. Proposed Method

This section outlines our innovative method for analyzing and bypassing the Heaven’s Gate technique. We present a two-fold methodology: first, we open the Heaven’s Gate using black-box testing to analyze the internal process of Heaven’s Gate, and then we close it to bypass Heaven’s Gate.

### 5.1. Opening Heaven’s Gate Using Black-Box Testing

We extract pertinent information from the Heaven’s Gate to investigate the internal execution process of the Heaven’s Gate technique. Initially, Heaven’s Gate was debugged using various analysis tools such as debuggers, disassemblers, and dynamic binary instrumentation (DBI). The debugging tools employed encompass Windbg [36], x64dbg [37], the Interactive Disassembler (IDA) Pro [38], and Pintool [39].

The only tool that can be executed inside Heaven’s Gate is the Windbg for a 64-bit program. However, its script features did not work in our testing. It appears that the script feature was not properly handled in the Heaven’s Gate, and as a result, we failed to use it. Although x64dbg for 32-bit program fails to trace 64-bit code after x64dbg Heaven’s Gate code individually, the x64dbg 32-bit version can execute the Heaven’s Gate at once. Given these constraints, we opted for black-box testing using x64dbg.

**Figure 1:**
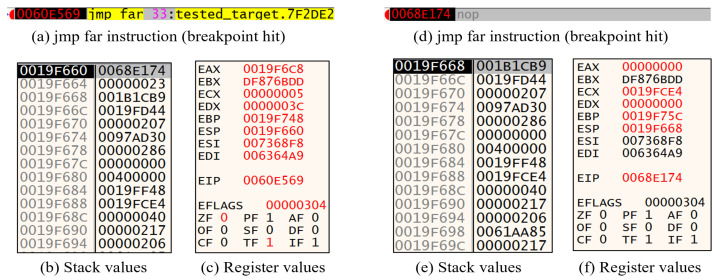
Comparative analysis of stack and register values before and after execution of HG.

To try black-box testing, we inserted a breakpoint at three types of instructions which can manipulate the CS selector: “jmp far”, “call far”, and “ret far”. Then, we executed the program and analyzed the environments, including register values, stack values, and page permissions, at the breakpoint using black-box testing. For example, Figure 1 shows stack and register values at the breakpoint hit. (a) in Figure 1 uses the jmp far 0x33:0x7F2DE2 instruction to enter Heaven’s Gate. This instruction modifies the value of the CS selector to 0x33 and jumps to the address 0x7F2DE2. Figure 1b shows that the stack address before Heaven’s Gate execution is located at 0x19f660, and the address memory value is 0x68E174. Furthermore, the value 0x23, which correlates to the Code Segment (CS) selector, is located at the subsequent memory address 0x19F664. Based on the information provided, it can be assumed that the function arguments of the Heaven’s Gate are given by the hexadecimal values 0x68E174 and 0x23.

**Figure 2:**
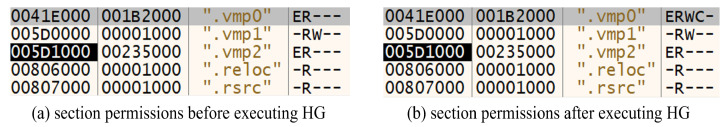
Comparative analysis of section permissions before and after execution of HG.

Since 0x23 corresponds to a 32-bit CS selector, it is reasonable to assume that the 32-bit address 0x68E174 with a 32-bit CS selector, is a 32-bit execution address. Therefore, a breakpoint was inserted at the address 0x68E174 and executed, as depicted in Figure 1d. After the execution, the stack value is observed as illustrated in Figure 1e. Moreover, a comparison of Figure 1b,f shows that the register values of EAX, ECX, EDX, EBP, ESP (equivalent to the stack address), and EIP (equivalent to the execution address location) have changed. From this result, it is observed that the 64-bit execution in the Heaven’s Gate technique results in an increment of the stack address by eight units. Additionally, it changes modifications in four register values, excluding the EIP and ESP register values, and subsequently moves to the address 0x68E174. Similarly, after executing the Heaven’s Gate, it is possible to examine the section permissions, as depicted in Figure 2. When analyzing the permissions in the section, both before and after the execution of the Heaven’s Gate, it is shown that the execution permissions for the .vmp0 section have changed from ER— to ERWC-. In summary, it can be assumed that the internal operation of the Heaven’s Gate consists of each register change and memory section change.

### 5.2. Closing Heaven’s Gate

When encountering a ‘far’ instruction, as described in Section 5.1, it is possible to eliminate the far instruction to change it to 64-bit execution. Subsequently, it is possible to move to the address after the execution of the Heaven’s Gate (address 0x68E174) and then call the function that operates within the Heaven’s Gate. The details of this algorithm are provided in Algorithm 1. In Algorithm 1, the HandleFarIns function handles far instructions before execution. Initially, the function identifies whether the given instruction, denoted by ins, is a far instruction by invoking the IsFarIns(ins) function.

If the instruction is identified as a far instruction, the algorithm computes the target address using the GetTargetAddress function. Subsequently, a validation phase is executed to ensure the validity of the targetAddress. If the targetAddress satisfies these criteria, the function performs a sequence of operations. Initially, it deletes far instructions using the DeleteInstruction function. Next, the BypassHG function reproduces the Heaven’s Gate code. It consists of a 32-bit function implementation that operates in the same manner as the 64-bit function used in Heaven’s Gate. Finally, it inserts a direct jump to the current address using the InsertDirectJump function. The implementation of the C/C++ code in the Pintool is presented in Listing 7.
**Algorithm 1:** Handling Far Instructions 1:**function** HandleFarIns(ins) 2:     **if** IsFarIns(*ins*) **then** 3:           *targetAddress* ← GetTargetAddress(*ins*) 4:           **if** targetAddress is valid **then** 5:                 DeleteInstruction(*ins*) 6:                 BypassHG(*ins*) 7:                 InsertDirectJump(*GetCurrentAddress(ins)*, *targetAddress*) 8:           **else** 9:                 HandleException()10:        **end if**11:    **end if**12:**end function**

The BypassHG function performs the same operations using 32-bit functions and register assignments. The details of this algorithm are provided in Algorithm 2. In Algorithm 2, the function starts by retrieving the current stack pointer, esp, and then incrementing it by eight bytes. This is executed to re-implement the previous changes between (b) and (e) in the previous Figure 1.
**Algorithm 2:** Reimplementation of Heaven’s Gate 1:**function** BypassHG 2:      *esp* ← GetCurrentStackPointer() 3:      IncreaseStackPointer(*esp*, 8) 4:      *processId* ← GetCurrentProcessID() 5:      *processHandle* ← OpenProcessWithAllAccess(*processId*) 6:      *scanAddress* ← ApplicationAddressLowerBound 7:      *lastSectionAddress* ← ApplicationAddressUpperBound 8:      **while** *scanAddress* < *lastSectionAddress* **do** 9:            ChangeMemoryProtection(*processHandle*, *scanAddress*, ExecuteWriteCopy)10:            *scanAddress* ← *scanAddress* + MemoryPageSize11:      **end while**12:      CloseProcessHandle(*processHandle*)13:**end function**

Subsequently, the current process ID is retrieved and used to obtain a handle to the process with all access rights. This handle is used to change the section permission. The scanAddress and lastSectionAddress variables are then initialized to the lower and upper bounds of the address space of application, respectively. A loop is then initiated that iterates over each memory page in the application’s address space, starting from scanAddress and ending at lastSectionAddress. For each memory page encountered, the memory protection is changed to the ExecuteWriteCopy function, which allows the code on that page to be executed, written, and copied. This is executed to re-implement the previous changes between (a) and (b) in the previous Figure 2. The handle to the process is closed after the loop has iterated over all pages in the application’s address space. The implementation of the C/C++ code in the pintool is presented in Listing 8.

**Listing 7.** Handling far jump to bypass Heaven’s Gate using DBI.

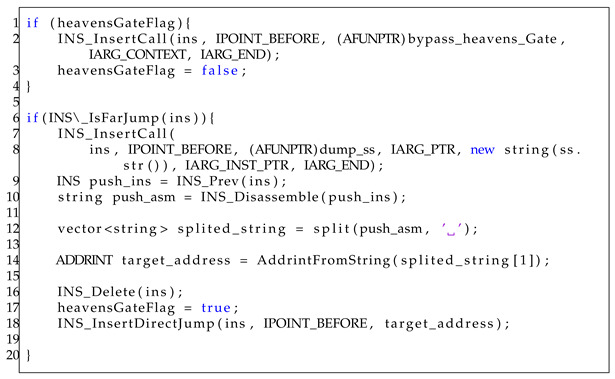



**Listing 8.** Bypass Heaven’s Gate using DBI.

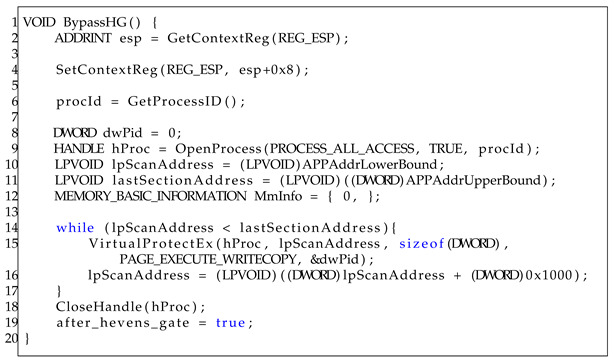



## 6. Evaluation

In this section, we present the experimental results of the proposed method. We measured the number of bypassed executable files using the proposed method. To demonstrate the effectiveness of the proposed method, we conducted experiments on various files using different input parameters and obtained the corresponding outputs. We implemented C++ code for the pintool and script-based code for x64dbg, then applied the Heaven’s Gate technique to the experimental files. To validate the practical application of our method, we employed the original entrypoint (OEP) search algorithm to check whether the actual OEP is reached. If our method functions correctly, the OEP is successfully reached; otherwise, Heaven’s Gate interrupts the OEP search, resulting in an error.

### 6.1. Dataset

For the experiment dataset, we collected an open-source dataset from CodeChef [40], a popular programming contest site. As CodeChef provides input samples for source code, we collected both of them for the experiment. Consequently, we collected 2616 C/C++ source codes and compiled the collected source codes using the GNU Compiler Collection (GCC) [41] with a 32-bit compile option. We obtained 2443 executable files except compile errors.

### 6.2. Implementation

We implemented the proposed method using pintool 3.22 and C/C++ libraries, as shown in Listings 7 and 8.

In Listing 7, the far jump instruction, which calls Heaven’s Gate, is deleted and replaced by the direct jump instruction. In Listing 8, the specific register targeted by VMProtect’s Heaven’s Gate mechanism was modified (line 4), and the permissions for the sections were subsequently altered (line 6–line 19).

### 6.3. Evaluation

We conducted our experiments on Windows 10 64-bit, equipped with an AMD Ryzen 9 3950X 16-Core Processor clocked at 3.49 GHz and 64 GB of RAM. The experiments were conducted using the following tools and versions: Pin tool v3.22, VMProtect v3.6 packer, x64dbg April 2022 version and the GNU’s Not Unix (GNU) Compiler Collection (GCC) v6.3.0 [41] for compiling the source codes. The source codes were compiled using a 32-bit compile option.

Our proposed method leveraged pintool and C/C++ libraries to implement the bypassing of VMProtect’s Heaven’s Gate mechanism. Pintool allowed us to instrument and analyze the execution of the target files, whereas our custom C/C++ libraries were used to manipulate the relevant registers and memory sections.

The experimental procedure involved two steps: packing and finding OEP. We initially packed 2443 32-bit files using VMProtect v3.6 with Heaven’s Gate. We applied the proposed bypass algorithm in Listings 7 and 8 OEP search algorithm through pintool and recorded the OEP when it was reached. We then verified if the recorded OEP matched the actual OEP. Out of 2443 files, our method successfully bypassed 2440 files. The three failure were caused by functional errors within the pintool. For the three failures, we manually applied the x64dbg bypass script in Listings A1 and A2 in Appendix A. We verified that the script codes bypassed Heaven’s Gate successfully.

To evaluate our algorithm’s effectiveness in actual malware analysis, we measured its execution time on the program being executed. From the experiment’s results, we found that Heaven’s Gate was performed, on average, eight times for each file. On average, the proposed bypass algorithm in Listings 7 and 8 required 0.00018 s to execute each file, resulting in a total execution time of 0.45539 s for 2440 files. Handling the far jump code in Listing 7 required 0.38982 s, while the bypassing process in Listing 8 took 0.06556 s. The longer execution time in processing the far jump code, compared to the bypass code, is due to comparing every instruction executed to identify if it was a far jump. On the other hand, the bypass code was executed an average of 8 times, a very small number of times compared to comparing all instructions. Algorithm 1 has a time complexity of O(1) because there is only linear execution, and Algorithm 2 has a time complexity of O(n) because it is repeated as many times as the number of sections. However, the number of program sections was a small constant, and the linear execution used in Algorithm 1 was performed a large number of times. As a result, Algorithm 1 took more time in the actual measurement.

These results indicate that our proposed method effectively bypasses VMProtect’s Heaven’s Gate mechanism, providing a robust solution for executing protected files, with a small overhead. This high success rate makes the proposed method a promising option for malware analysts and security researchers. Notably, the 36 file failures were not attributed to our proposed method but rather to issues during the unpacking process, which fall outside the scope of our research.

## 7. Limitation and Future Work

Based on the experimental results, the proposed method was successful in all scenarios. However, our proposed method was tested on the latest version of VMProtect only because it is the only tool that uses Heaven’s Gate. Although the proposed method has currently been tested only on VMProtect, its black-box testing approach is also adaptable to other variants. Therefore, even if the Heaven’s Gate technique is used in other tools in the future, it can be bypassed through the information obtained through the proposed black-box testing. Additionally, since Heaven’s Gate is not covered in other anti-debugging related studies, there is a problem of a lack of comparative anti-debugging research. To the best of my knowledge, Heaven’s Gate has not been addressed before.

It is also possible pin tool might become unavailable or incompatible with future systems. To mitigate it, alternative DBI tools can be employed such as DynamoRIO [42] and Valgrind [43]. Also, the debugger can be used as described in Listings A1 and A2 in the Appendix. We conducted experiments only on C/C++ based programs in the Windows OS environment. Future work should focus on enhancing the robustness of the proposed method on other OSs and languages. Future work will also include an analysis of new technologies related to anti-debugging that could be combined with Heaven’s Gate and ways to circumvent them.

## 8. Conclusions

The increasing sophistication of malware techniques presents a continuous challenge to existing security measures, including DBI tools, which are crucial for various applications, such as program analysis, unpacking, and de-virtualization. The Heaven’s Gate technique, which exploits the switching between 32-bit and 64-bit modes during execution, is particularly challenging as it not only bypasses security software designed for 32-bit processes but also presents an opportunity for attackers to exploit DBI tools. In this paper, we have provided a comprehensive analysis of the Heaven’s Gate technique on high-level language and proposed a novel approach using black-box testing. We mitigated this problem using the DBI tool and debugger script. Our experimental results demonstrated the effectiveness of the proposed approach in bypassing the Heaven’s Gate technique against advanced malware. Our algorithm bypassed Heaven’s Gate in 0.00018 s per file, totaling 0.45539 s for 2440 files with a low overhead. Our research contributes significantly to the domain of cyber security, offering a robust and rapid-response mechanism against malware threats.

## Figures and Tables

**Table 1:** Summary of Related Work.

Reference	Scope	Analysis Tools	Methodology
Apate [26]	Anti-debugging	WinDbg	JIT disassembling based on singlestepping
Lee et al. [19]	Anti-debugging	Pin	A detailed analysis of anti-analysis techniques
EvDetector [31]	Anti-debugging	Pin	System checks, user activity-based checks
HybridEmu [32]	Anti-debugging	Bochs emulator	A CPU simulator, actual code execution
Park et al. [27]	Anti-debugging	Pin	Matching suspicious code chunk and specific anti-debugging technique
PinDemonium [20]	Unpacking	Pin, Scylla	WxorX and heuristics methods, IAT fixing using scylla
Arancino [22]	Unpacking		Anti-instrumentation techniques based on PinDemonium
BinUnpack [29]	Unpacking	Not available	Kernel-level DLL hijacking
x64Unpack [28]	Unpacking	Bochs emulator	A CPU simulator, actual code execution
Proposed Method [28]	Anti-debugging	Pin, x64dbg	Black-box testing

## Data Availability

The data presented in this study are openly available at (https://github.com/unlockable/Bypassing-Heaven-s-Gate, accessed on 23 November 2023).

## Abbreviations

The following abbreviations are used in this manuscript:

WOW64	Windows-on-Windows 64-bit subsystem
DBI	Dynamic Binary Instrumentation
IoT	Internet of Things
DLL	Dynamic Linked Library
CS	Code Segment
PE	Portable Executable
OEP	Original Entry Point
IAT	Import Address Table
HG	Heaven’s Gate
GNU	GNU’s Not Unix
GCC	GNU Compiler Collection
IDA	The Interactive Disassembler
API	Application Programming Interface

## Appendix A

**Listing A1.** HHandling far jump to bypass Heaven’s Gate using DBG.

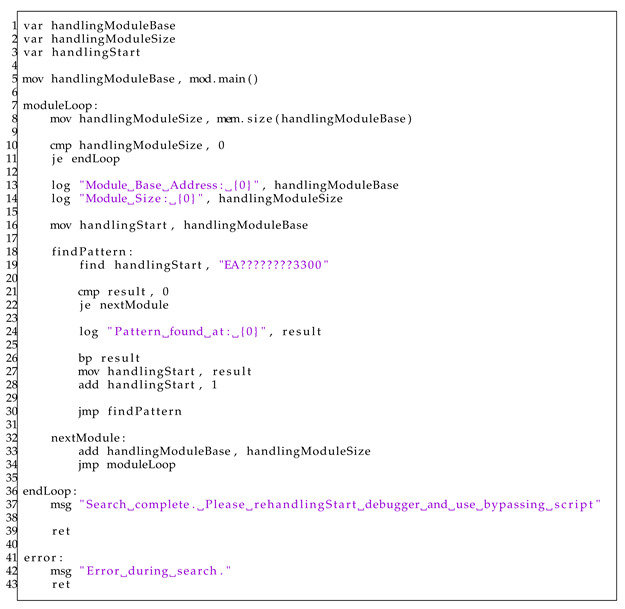

**Listing A2.** Bypass Heaven’s Gate using DBG.

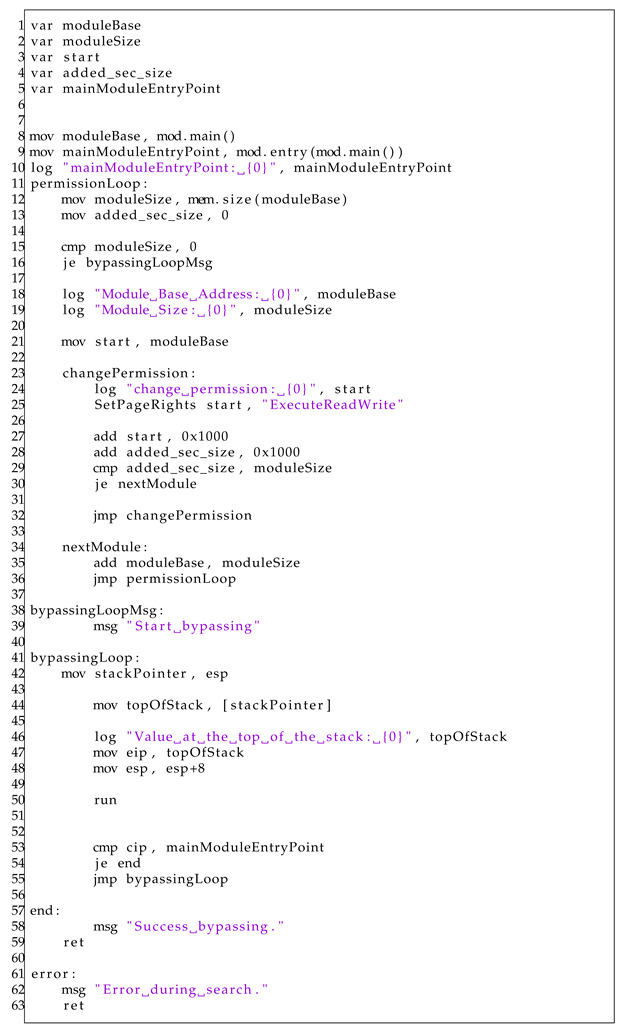

Listings A1 and A2 show the implementation of the proposed algorithm in the x64dbg script. Listing A1 searches for far commands entering Heaven’s Gate and sets breakpoints. Then, after restarting the debugger, Listing A2 performs a Heaven’s Gate bypass. In this process, the permission of the section is changed, and the value of esp is changed.
